# Valve-in-Valve Therapy for the Intrepid Mitral Valve First-in-Human Report of Acute and Chronic Prosthesis Management

**DOI:** 10.1016/j.shj.2023.100184

**Published:** 2023-05-06

**Authors:** Paul Sorajja, João L. Cavalcante, Richard Bae, Vinayak N. Bapat

**Affiliations:** Valve Science Center, Minneapolis Heart Institute Foundation, and the Allina Health Minneapolis Heart Institute at Abbott Northwestern Hospital, Minneapolis, Minnesota, USA

**Keywords:** Intrepid, Mitral regurgitation, Valve-in-valve

Transcatheter mitral valve replacement (TMVR) is an emerging therapy for mitral regurgitation (MR)^1^, ^2^, ^3^. While the initial experience has been favorable, repeat therapy may be required in some instances. These instances include acute occurrences, such as device malposition, as well as chronic prosthesis degeneration.

The Intrepid TMVR prothesis (Medtronic Inc., Galway, IR) is a unique therapy, composed of a self-expanding, nitinol double-frame, which houses a 27 mm tri-leaflet bovine valve. As a relatively new technology, little is known about its ability to accommodate repeat mitral intervention, especially with regard to possible transcatheter mitral valve-in-valve therapy.

Herein, we describe the first 2 human cases of transcatheter mitral valve-in-valve therapy with the Intrepid TMVR prosthesis. We describe considerations and techniques that are specific to the clinical indications for such valve-in-valve therapy, with a description of patients treated in acute and chronic settings.

## Acute Device Malposition

**Figure 1:**
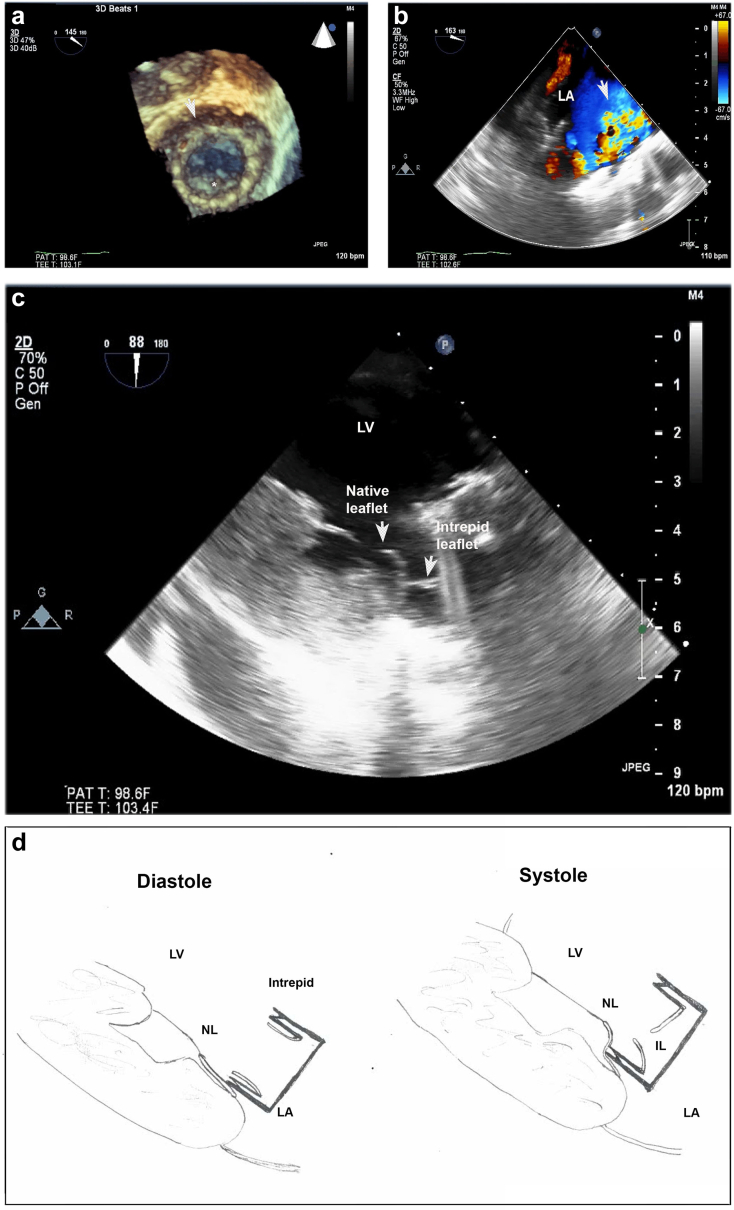
**Acute mitral regurgitation due to acute malposition and leaflet overhang with an Intrepid prosthesis.** (a) Following deployment of the Intrepid prosthesis (arrowhead), there was immobility of one of the prosthetic leaflets (asterisk). (b) Acute severe mitral regurgitation was present (arrowhead). (c). The mechanism of immobility was atrial malposition, leading to native leaflet overhang. (d). Illustration of the mechanism of immobility from atrial malposition in diastole and systole. Abbreviations: IL, Intrepid leaflet; LA, left atrium; LV, left ventricle; NL, native leaflet.

**Figure 2:**
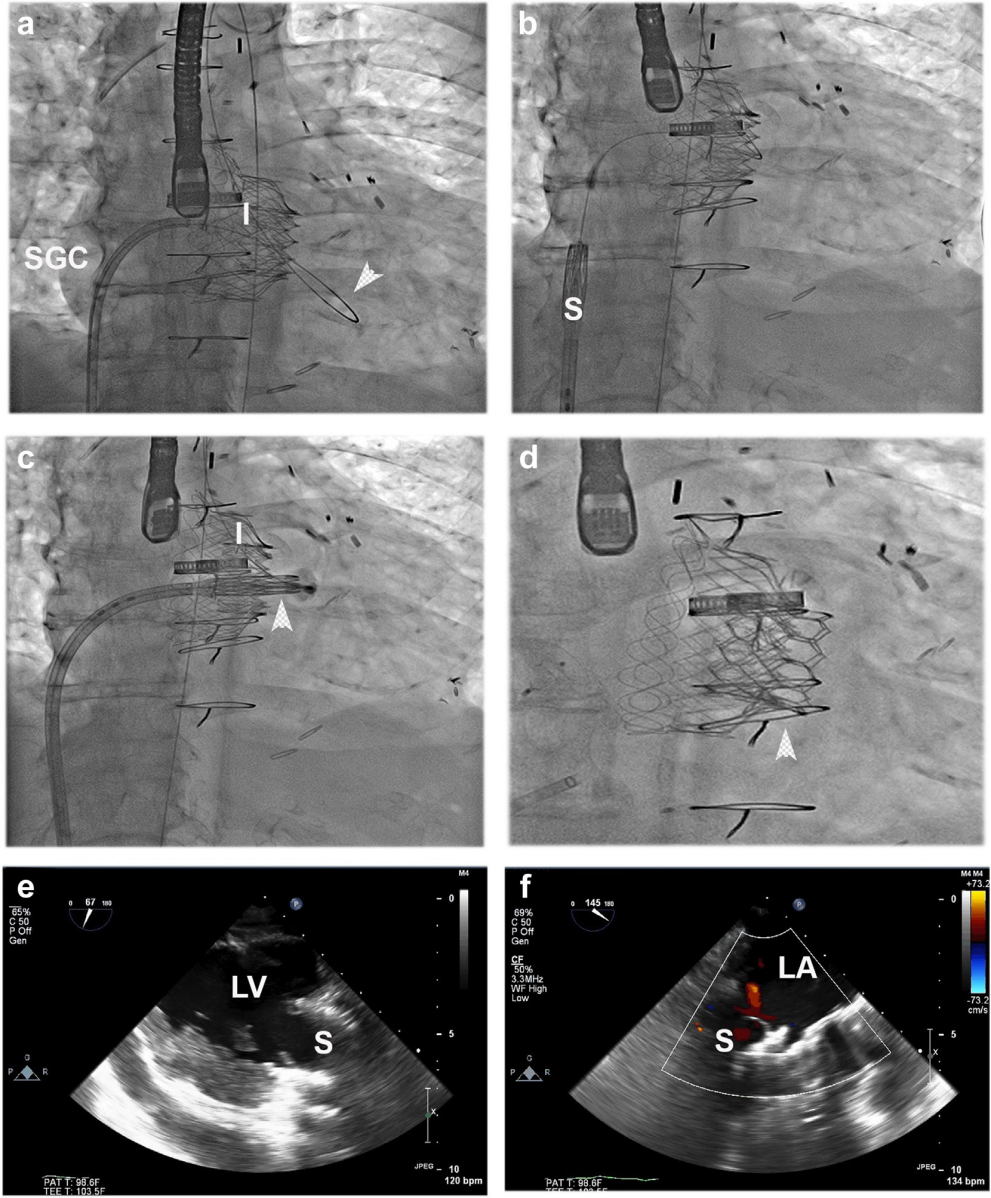
**Mitral valve-in-valve therapy for acute malposition of the Intrepid prosthesis.** (a) To minimize the risk of movement of the Intrepid prosthesis that could lead to inversion, a rail (arrowhead) was created using a steerable guide catheter (SGC). (b) Tension on the rail helps to ensure passage of the Sapien (S) prosthesis without affecting the Intrepid valve. (c) and (d) To treat leaflet overhang, the ventricular side of the Sapien prosthesis was positioned one-third from the Intrepid outflow portion (arrowheads). (e) and (f) Echocardiography showed coverage of the native leaflet with the Sapien prosthesis and elimination of the MR. Abbreviations: LA, left atrium; LV, left ventricle; MR, mitral regurgitation.

The patient was a 75-year-old man with severe, symptomatic MR, with ischemic cardiomyopathy (ejection fraction, 40%) and prior coronary artery bypass grafting. After a full heart team evaluation, he was enrolled in the Twelve (Intrepid) TMVR Pilot Study (NCT02322840). Following a left thoracotomy, a 50 mm Intrepid prosthesis was delivered transapically and deployed with rapid ventricular pacing. Postimplantation, transesophageal echocardiography (TEE) demonstrated severe transvalvular regurgitation, which was due to atrial malposition and native leaflet overhang across the prosthetic leaflets (Figure 1). An intra-aortic balloon pump was placed for hemodynamic stability. After a full discussion of alternatives, mitral valve-in-valve therapy with a Sapien S3 (Edward Lifesciences, Irvine, CA) was undertaken (Figure 2).

Transseptal puncture from the right femoral vein was performed with a C0 Baylis catheter at a mitral height of 4.5 cm, followed by exchange for an 8.5 Fr Agilis catheter (Abbott Vascular, Santa Clara, CA). In order to minimize the risk of movement of the newly implanted Intrepid prosthesis, a rail was created with a Roadrunner wire that was snared by a 15 mm gooseneck in the descending aorta, and exteriorized via the right femoral artery. The atrial septum was dilated with a 14 Fr Inoue dilator, followed by the placement of a 16 Fr expandable sheath in the femoral vein. A distance of 8 mm from the outflow of the Intrepid prosthesis frame to cover the leaflet overhang was measured on TEE. With an expected final frame height of 22.5 mm for the 29 Sapien 3, the Sapien 3 prosthesis was positioned with one-third extending from the Intrepid outflow. There was complete elimination of leaflet overhang, relief of MR, no mitral stenosis (i.e., gradient, 2 mmHg), and no left ventricular outflow tract obstruction (i.e., gradient, 6 mmHg). The atrial septum was then closed with a 10 mm Amplatzer occluder.

## Chronic Prosthesis Degeneration

A 72-year-old man with severe MR due to nonischemic cardiomyopathy (ejection fraction, 40-45%) was treated with successful, transapical placement of a 46 mm Intrepid prosthesis as part of the Intrepid TMVR Pilot Study (NCT02322840). During follow-up visits for 5 years after the implantation, he was asymptomatic, with normal prosthetic function. On a routine examination at 5.5 years, however, a loud murmur was heard and subsequent echocardiography demonstrated recurrent severe regurgitation with a mean mitral gradient of 14 mmHg. New-onset heart failure occurred 1 ​month later, and a TEE demonstrated a flail prosthetic leaflet in the Intrepid valve. Cardiac computed tomography confirmed the neo-left ventricular outflow tract was unchanged from prior to Intrepid implantation.

**Figure 3:**
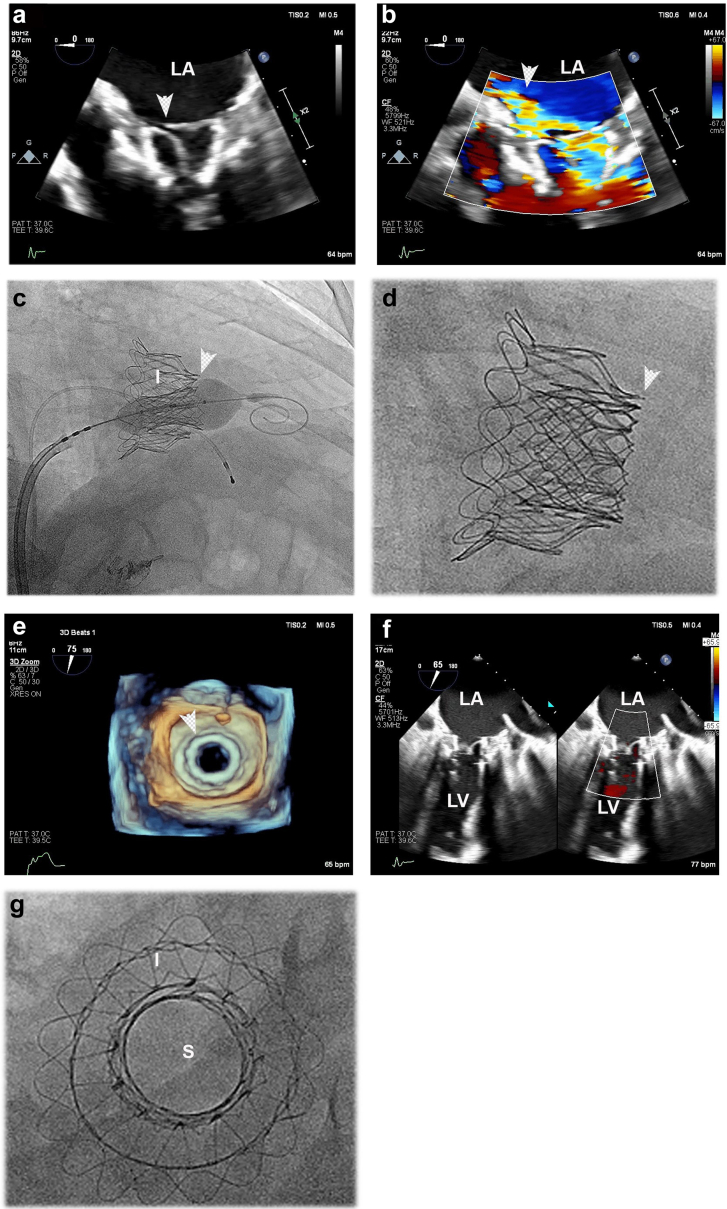
**Mitral valve-in-valve therapy for an Intrepid prosthesis with chronic degeneration.** (a) Transesophageal echocardiography (TEE) shows a flail segment (arrowhead) in a patient who had Intrepid implantation 5.5 years previously. (b) Severe mitral regurgitation (MR) is present (arrowhead). (c) and (d) A 26 mm Sapien valve is placed with matching of the outflow frames of the 2 prostheses (arrowheads). (e) The Sapien (arrowhead) is fully expanded. (f) Complete relief of MR occurs. (g) En face view of the final valve-in-valve implantation. Abbreviations: I, Intrepid prosthesis; LA, left atrium; LV, left ventricle; S, Sapien prosthesis.

For this patient, mitral valve-in-valve therapy was performed using a transseptal approach and matching of the outflow frames for the Intrepid and a 26 mm Sapien 3 prosthesis (Figure 3). An inferior-posterior transseptal puncture at a height of 5.3 cm was used. Given the chronic nature of the Intrepid implantation, the mitral valve-in-valve procedure was performed only with a 260 cm Safari XS wire in the left ventricle (i.e., no rail). There was complete relief of the MR, with a final mean gradient of 2 mmHg (heart rate, 81 beats per minute). A chronic pericardial effusion also was treated with pericardiocentesis and removal of 750 ml at the conclusion of the procedure.

## Conclusions

Mitral valve-in-valve therapy in the setting of prior Intrepid TMVR therapy needs to be performed with tailoring of the implantation according to the pathology presented. With multimodality imaging assessment for procedural planning, such procedures can be effective in relieving acute or chronic MR.

## Consent Statement

Consent was obtained from the patient for publication of this report and any accompanying images.

## Funding

The authors have no funding to report.

## Disclosure Statement

Dr Cavalcante has received consulting fees from 4C Medical, Abbott Structural, Anteris, AriaCV, Boston Scientific, Edwards Lifesciences, JenaValve, Medtronic, VDyne, WL Gore, Xylocor; has received research grant support from 10.13039/100016262Abbott Northwestern Hospital Foundation and Abbott Structural, and has served on Advisory Boards for Abbott Structural, Boston Scientific, Medtronic. Dr Sorajja has received consulting fees from 4C Medical, Abbott Structural, Medtronic, Boston Scientific, Edwards Lifesciences, Anteris, W.L. Gore; institutional research grants from Abbott Structural, 10.13039/100004374Medtronic, and 10.13039/100008497Boston Scientific, and a speaker for Abbott Structural. Dr Bae has received consulting fees from Abbott Structural. Dr Bapat has received consulting fees from Medtronic.
